# How Cannabis Causes Paranoia: Using the Intravenous Administration of ∆^9^-Tetrahydrocannabinol (THC) to Identify Key Cognitive Mechanisms Leading to Paranoia

**DOI:** 10.1093/schbul/sbu098

**Published:** 2014-07-16

**Authors:** Daniel Freeman, Graham Dunn, Robin M. Murray, Nicole Evans, Rachel Lister, Angus Antley, Mel Slater, Beata Godlewska, Robert Cornish, Jonathan Williams, Martina Di Simplicio, Artemis Igoumenou, Rudolf Brenneisen, Elizabeth M. Tunbridge, Paul J. Harrison, Catherine J. Harmer, Philip Cowen, Paul D. Morrison

**Affiliations:** ^1^Department of Psychiatry, University of Oxford, Oxford, UK;; ^2^Centre for Biostatistics, Institute of Population Health, University of Manchester, Manchester, UK;; ^3^Department of Psychosis Studies, Institute of Psychiatry, King’s College London, London, UK;; ^4^Department of Computer Science, University College London, London, UK;; ^5^Institució Catalana de Recerca i Estudis Avançats (ICREA), University of Barcelona, Barcelona, Spain;; ^6^Oxford Health NHS Foundation Trust, Oxford, UK;; ^7^Waikato Hospital, Hamilton, New Zealand;; ^8^MRC Cognition and Brain Sciences Unit, Cambridge, UK;; ^9^Queen Mary University of London, Violence Prevention Research Unit, Barts and the London School of Medicine and Dentistry, Wolfson Institute of Preventive Medicine, London, UK;; ^10^Department Clinical Research, University of Bern, Bern, Switzerland

**Keywords:** paranoia, delusions, cannabis, THC, cognitive

## Abstract

Paranoia is receiving increasing attention in its own right, since it is a central experience of psychotic disorders and a marker of the health of a society. Paranoia is associated with use of the most commonly taken illicit drug, cannabis. The objective was to determine whether the principal psychoactive ingredient of cannabis—∆^9^-tetrahydrocannabinol (THC)—causes paranoia and to use the drug as a probe to identify key cognitive mechanisms underlying paranoia. A randomized, placebo-controlled, between-groups test of the effects of intravenous THC was conducted. A total of 121 individuals with paranoid ideation were randomized to receive placebo, THC, or THC preceded by a cognitive awareness condition. Paranoia was assessed extensively via a real social situation, an immersive virtual reality experiment, and standard self-report and interviewer measures. Putative causal factors were assessed. Principal components analysis was used to create a composite paranoia score and composite causal variables to be tested in a mediation analysis. THC significantly increased paranoia, negative affect (anxiety, worry, depression, negative thoughts about the self), and a range of anomalous experiences, and reduced working memory capacity. The increase in negative affect and in anomalous experiences fully accounted for the increase in paranoia. Working memory changes did not lead to paranoia. Making participants aware of the effects of THC had little impact. In this largest study of intravenous THC, it was definitively demonstrated that the drug triggers paranoid thoughts in vulnerable individuals. The most likely mechanism of action causing paranoia was the generation of negative affect and anomalous experiences.

## Introduction

Paranoia—unfounded fears that others intend harm to the individual—is a central experience of psychotic disorders such as schizophrenia. Factor analytic studies indicate that it is an independent experience that requires explanation in its own right.^1^ Many people have a few paranoid ideas, and a few people have many.^2^ Paranoia is associated with youth, poverty, poor physical health, suicidal ideation, and the use of cannabis.^3^ We took this latter association to carry out a unique experimental investigation into the causes of paranoia. First, we set out to establish that cannabis causes paranoia, using the most comprehensive battery ever used to assess such fears. Second, cannabis was used as a probe to identify the key cognitive mechanisms causing paranoid fears. Determination of the immediate causes can be used to advance the treatment of delusions.^4^

### A Cognitive Account of Paranoia

**Fig. 1.**
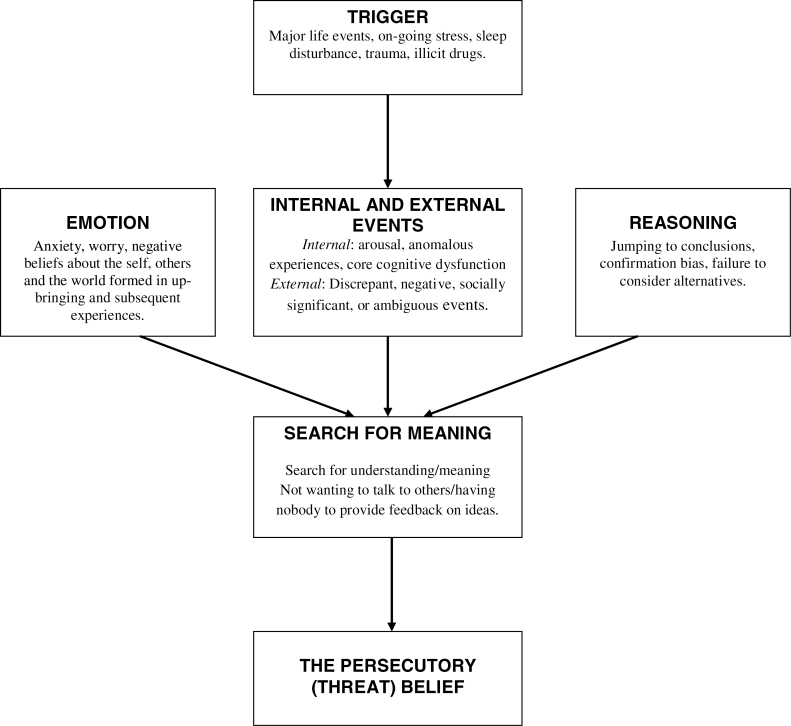
Outline of factors involved in persecutory delusion development.^5^

Our cognitive account of paranoia identifies multiple causes (see figure 1).^5^ It is hypothesized that the individual experiences an anomalous internal state. These anomalies may include, across the different senses, changes in sensory intensity, distorted sensory experience, sensory flooding, unusual sensory experience, thought echo, and hallucinations.^6^ They may also include feelings of unusual salience.^7^ The anomalies may be triggered by, eg, life events, poor sleep, or illicit drugs such as cannabis. In essence, the person feels *different* and this requires an explanation. Odd experiences encourage unusual thoughts. Importantly, a negative affective state makes a paranoid interpretation likely: anxiety leads to the threat content; negative self beliefs highlight the person’s vulnerability to harm; and engagement in worry results in negative, implausible ideas. The fears reach a delusional level of conviction when reasoning biases, such as jumping to conclusions, are present. Working memory performance moderates the effect of the reasoning biases.^8,9^

### Using ∆^9^-Tetrahydrocannabinol to Determine the Causes of Paranoia

Establishment of causal roles requires manipulation of the factors of interest.^10^ We saw the administration of cannabis as a method of manipulating key putative causal factors in paranoia, while also providing an important opportunity to learn about a substance seen by many as a contributory cause of psychosis (eg, Casadio et al^11^).

The principal psychoactive constituent of cannabis is ∆^9^-tetrahydrocannabinol (THC). THC administered intravenously is characterized by the appearance of psychopharmocological effects within 5 minutes, which continue for at least 90 minutes, providing an excellent experimental window.^12^ In within-subjects tests with nonclinical volunteers, D’Souza, in particular, has shown that intravenous administration of THC causes schizophrenia-like symptoms, perceptual disturbances, anxiety, and impaired working memory (eg, Morrison et al^13^ and D’Souza et al^14^,^15^). Similar but more pronounced results were found in patients with schizophrenia.^15^

Principally, the psychosis-inducing effects of cannabis have been linked to an abnormal salience theory,^16,17^ in which THC leads to an increase in anomalous experience which is then misinterpreted. However, THC can also induce anxiety via cannabinoid receptors in the amygdala.^18^ Therefore, the prediction from the cognitive model of paranoia is that THC leads to such fears via 2 routes: the generation of confusing anomalous experiences and an increase in negative affect and related processes. Working memory is clearly affected by THC, but since such cognitive problems have not been linked to the positive symptoms of psychosis,^19^ a direct memory performance route to paranoia would not be expected.

### The Current Study

In the current study, we set out to identify the causes of paranoia via the administration of THC. This requires a between-groups test of the administration of THC against placebo. A third condition tested a further causal factor: misinterpretation. The cognitive perspective considers paranoia to be a misinterpretation of events. If individuals are made sufficiently aware of the potential subjective effects of THC, then this may alter the interpretation made and hence the occurrence of paranoia. Therefore, the third condition was a cognitive awareness manipulation in which the potential effects of THC were explained before drug administration. The participants to be tested were selected from the general population on the key criterion of having had recent paranoid ideation. This was therefore an analogue population, in order that the results are applicable to understanding the clinical phenomenon; testing individuals without signs of vulnerability to disorder would be much less informative about clinical paranoia, while testing patients in such large numbers would have ethical and practical difficulties. We were mindful of “the paranoia problem,” the difficulty of determining whether persecutory ideation is unfounded, so the battery of tests included the most extensive range of paranoia assessments yet used in a study. There were 3 hypotheses: (*a*) THC increases the occurrence of paranoia, (*b*) the occurrence of anomalous experiences and negative affect (but not alterations in working memory) explains (ie, mediates) the increase in paranoia, and (*c*) that paranoid interpretations can be partially blocked by cognitive awareness. The second hypothesis concerning underling mechanisms is the hardest to establish—we note the advice of Bullock and colleagues^20^ “to think of mediation analysis as a cumulative enterprise.”

## Methods

### Participants

There were 121 participants. The inclusion criteria were: aged between 21 and 50, had taken cannabis at least once before, and reported a paranoid thought in the past month as assessed by the Paranoid Thoughts Scale Part B.^21^ The exclusion criteria were: a history of mental illness or substance dependence, a history of major mental illness in a first-degree family member, a neurological condition, a heart condition, a history of fainting, photo-sensitive epilepsy, or being a pregnant or nursing mother. The inclusion criteria concerning age and previous use of cannabis, as well as all the exclusion criteria, were designed to minimize the chances of any significant adverse effects. The psychiatrists carrying out the administration of the vials also carried out a brief health check. The study had received approval from an NHS research ethics committee, and written informed consent was received from all participants. Participants were recruited via distribution of leaflets to local postcodes and the playing of local radio adverts in Oxfordshire, UK.^22^ The study was also advertised at a number of colleges in the University of Oxford, at Oxford Brookes University, and on the website of a local newspaper. The adverts did not mention cannabis or paranoia. The wording was: “Volunteers Required for Psychological Research. We are looking for volunteers to take part in a medical research study being carried out at the university. The research would take three hours and you would be compensated for your time. If you would like to hear more about the research, then please contact us. We send detailed information about the study so that you can consider whether you would like to take part.” The individuals who responded were then invited to take part in the screening stage. Depending on participant preference, the screening questionnaires were either completed online via a web-link or sent via post. Therefore, screening was self-report. A total of 1792 people were screened. The reasons for exclusion (which could be several for a person) were: outside the age range (*n* = 611), had not taken cannabis before (*n* = 1117), did not report persecutory ideation (*n* = 739), had a history of mental illness (*n* = 281), substance abuse (*n* = 41), or major mental illness in a first-degree family member (*n* = 100), had a neurological condition (*n* = 37), had a history of fainting (*n* = 53), had a heart condition (*n* = 83), had photo-sensitive epilepsy (*n* = 2), were pregnant or a nursing mother (*n* = 17), had other physical health problems (*n* = 5), or had high blood pressure prior to administration of the vial (*n* = 5). Totally, 106 people were suitable but declined to take part or were not contactable.

### Design

The study was a between-groups design. After assessment on the cognitive variables, participants were randomized to either placebo, THC, or THC with cognitive awareness. Randomization was carried out by a researcher independent of recruitment and testing, using randomized permuted blocks of varying size with a plan created from www.randomization.com. Participants, assessors, and the psychiatrists were blind to the placebo and THC conditions. For the cognitive awareness condition, both participant and assessor were aware the participant was to receive THC. After administration of the contents of the vials, participants completed the paranoia assessments and repeated the cognitive tests. Testing on all measures postadministration was complete in an average of 83 minutes (SD = 18). There was a debriefing at the end of testing. A taxi was provided for returning participants home at the end. Participants were contacted the following day to check for any adverse effects.

### Randomization Conditions

THC (Dronabinol) was supplied by THC Pharm GmbH and prepared as vials for injection by Bichsel Laboratories according to the method of Naef and colleagues.^23^ The THC had 99.5% purity. A 1.5 mg dose of THC was used. Vials of the placebo and THC were indistinguishable. A psychiatrist administered either placebo (10 ml of saline) or synthetic THC (1.5ml of THC diluted in saline to 10 ml volume). The solutions were administered via an indwelling forearm cannula in 1 ml pulses every 1 minute for 10 minutes. Blood pressure was monitored during this period, and the psychiatrist was always available during the testing. Lorazepam (oral, 1–2 mg) was available in case a person became significantly distressed but this was not used during the study.

The cognitive awareness condition, given before THC administration, involved a simple 5-minute educational module, explaining the range of effects that the drug can cause (THC was considered synonymous with cannabis for this procedure). From before to after the awareness training, ratings on 2 visual analogue scales significantly increased; these assessed the participants’ potential attribution of effects (“After being given the cannabis, how much do you believe that if you have any good or bad feelings over the next 90 minutes that cannabis will be the cause?”), *t*(40) = −6.59, *P* < .001, and confidence to keep this in mind (“If you feel different after being given cannabis, how easy will it be to keep in your mind that the cannabis is the cause?”), *t*(40) = −5.11, *P* < .001.

### Assessments

The National Statistics Socioeconomic Classification analytic classes were used.^24^ History of illicit drug use was collected using the Maudsley Addiction Profile.^25^ Intellectual functioning was estimated using the Wechsler Abbreviated Scale of Intelligence.^26^

Paranoia was assessed in 4 ways. A total score (Paranoid VAS) was obtained from 6 visual analogue scales (“Right now I feel suspicious of other people,” “Right now I feel that people want to harm me,” “Right now I feel like people want to upset me,” “Right now I feel like people are against me,” “Right now I am thinking that others are trying to persecute me,” and “Right now I feel like people are being hostile towards me”) (Cronbach’s α = .90), which were used at baseline, after administration of the vial contents, and at the end of testing. Immediately after vial administration, participants were escorted on a 5-minute walk to the virtual reality testing room. On the way, they were asked to go into the hospital canteen and purchase an item. After doing this, they completed the 6 paranoia visual analogue scales in relation to the people encountered on the walk (Social Situation Paranoia). They then completed 5 minutes in an immersive virtual reality social environment (see supplementary figure 1). The virtual underground was displayed using an NVIS SX111 head mounted display (as used in Freeman et al^27^). Because the environment is neutral, any perceived hostility is known to be unfounded. Participants’ views of the computer characters were assessed using the State Social Paranoia Scale (SSPS)^28^ and a visual analogue scale (“Please mark on the line how hostile you thought the people on the tube were”) (VAS VR Hostile). Participants were also assessed by an interviewer on the suspiciousness item of the Positive and Negative Symptom Scale (PANSS Suspiciousness).^29^ The Community Assessment of Psychic Experiences (CAPE)^30^ was included, completed for the time period since vial administration.

The following putative mediation variables were assessed: anomalous experiences using the Cardiff Anomalous Perceptions Scale (CAPS)^6^; anxiety using the Beck Anxiety Inventory^31^; visual analogue scales for anxiety (“how anxious you feel right now”), depression (“how miserable or sad you feel right now”), worry (“how worried you feel right now”), and self-focus (“Right now my attention is focused on my inner thoughts and feelings,” “Right now my attention is focused on how I appear to others,” “Right now my attention is focused on my surroundings”); catastrophizing^32^; interpersonal sensitivity^33^; negative and positive views of the self and others using the Brief Core Schema Scales^34^; threat anticipation^35^; jumping to conclusions^36^; belief flexibility assessed by responses indicating that there is a possibility that participants could be mistaken in 3 high conviction beliefs; and working memory assessed by the digit span and letter-number subtests of the WAIS III.^37^ Questionnaires were adapted for state use as appropriate.

### Statistical Analysis

All analyses were carried out using Stata^38^ and SPSS.^39^ The analyses involved data reduction (simplification) using principal components analyses (PCA), our aim being to generate orthogonal (uncorrelated) composite measures to eliminate the problems of colinearity. No attempt was made to fit a formal measurement model (eg, via confirmatory factor analysis), the latter being both unnecessary and inappropriate for an exploratory analysis of the present type. Instead of doing multiple analyses of the paranoia outcomes, a PCA was undertaken (on the correlation matrix) for all 6 measures together (Paranoid VAS post THC administration, Paranoid VAS at end of testing, Social Situation Paranoia, SSPS, VAS VR Hostile, and PANSS Suspiciousness) and the first principal component (representing 71% of the total variation) was used as the primary outcome in all future analyses. This component was scaled to have a mean (for the whole sample) of 0 and a SD of 1 (note that the scaling is arbitrary and has no effect on the findings). The following weights were used to calculate this first principal component from the above 6 standardized paranoia assessments, respectively: 0.433, 0.428, 0.350, 0.417, 0.41, and 0.398. Principal component scores were available for 119 participants.

Two binary dummy variables were then created: THC = 0 for placebo, =1 for THC or THC + Cognitive awareness; Cognitive Awareness = 0 for placebo or THC, =1 for THC+ Cognitive Awareness. When both are entered together as explanatory variables in a regression model, then the parameter corresponding to THC is the estimate of receiving THC and the parameter for Cognitive Awareness is the effect of the cognitive awareness condition on those receiving THC. Intention-to-treat (ITT) effects were then estimated using linear regression (ANCOVA). The initial visual analogue scales for paranoia and for anxiety were both used as baseline covariates to increase precision.

Similar to the data reduction exercise for the paranoia measures, all of the 27 mediation variables were first entered into a PCA, resulting in 8 principal components with an eigenvalue greater than 1.0 (the well-known Kaiser criterion—explaining 30.1%, 9.2%, 7.2%, 6.1%, 5.5%, 5.0%, 4.2%, and 3.9% of the total variation, respectively). These components were then subjected to an orthogonal (Varimax) rotation for ease of interpretation. The rotated components were then used as the mediators. The main variables contributing to these rotated components were: anomalous experiences, anxiety, worry, depression and negative beliefs about the self (component 1); internal self-focus of attention (component 2); working memory (component 3); external focus of attention (component 4); interpersonal sensitivity (component 5); belief flexibility and positive beliefs about self and others (component 6); threat anticipation (component 7); and catastrophizing and jumping to conclusions (component 8). Full details of the components are provided in supplementary table S1. Component scores were available for 114 participants.

Mediation was tested using regression methods following the logic of Baron and Kenny.^40^ There were three steps to determine mediation: an ITT effect of randomization condition on the outcome (paranoia); an ITT effect of randomization condition on the putative mediator; and when the outcome (paranoia) is jointly predicted by both the randomization condition and mediator, the mediator effect is significant and the randomization condition effect is lowered. In these regressions, the binary dummy variables THC and Cognitive Awareness were entered together to test the effects of the randomization conditions, and the initial visual analogue scales for paranoia and for anxiety were used as baseline covariates.

Acknowledging that we are primarily carrying out an exploratory analysis, we did not alter significance levels for multiple testing, agreeing with the view that “simply describing what tests of significance have been performed, and why, is generally the best method of dealing with multiple comparisons.”^41^ Data on all outcomes was available for 114 participants and with such small amounts of missing data, this is unlikely to be a cause of any significant biases. All significance testing was two-tailed.

## Results

The basic demographic data for the participant groups can be seen in table 1.

**Table 1.** Composition of the Three Randomization Groups

	Placebo	THC	THC + Awareness
	(*n* = 41)	(*n* = 41)	(*n* = 39)
Mean age in years (SD)	30.3 (9.6)	30.8 (8.5)	28.0 (6.8)
Gender			
Male	30	26	25
Female	11	15	14
Ethnicity			
White	38	36	37
Black African	0	1	0
Black other	0	0	1
South Asian	0	1	1
Other	3	3	0
Socioeconomic status			
Higher professional	2	5	0
Lower professional	4	6	6
Intermediate	5	5	4
Own account workers	1	6	1
Lower supervisory	4	1	4
Semi-routine	6	3	4
Routine	2	4	6
Long-term unemployed	3	2	3
Student	14	9	11
IQ (SD)	116.7 (10.8)	114.1 (12.9)	114.0 (11.5)
Number of times used cannabis in the past month (SD)	2.2 (5.8)	4.5 (12.7)	5.6 (17.3)
Paranoid Thoughts Scale Part B at screening (SD)	25.1 (10.6)	23.9 (9.4)	27.8 (11.6)

*Note:* THC, Δ_9_-tetrahydrocannabinol.

### Paranoia

**Fig. 2.**
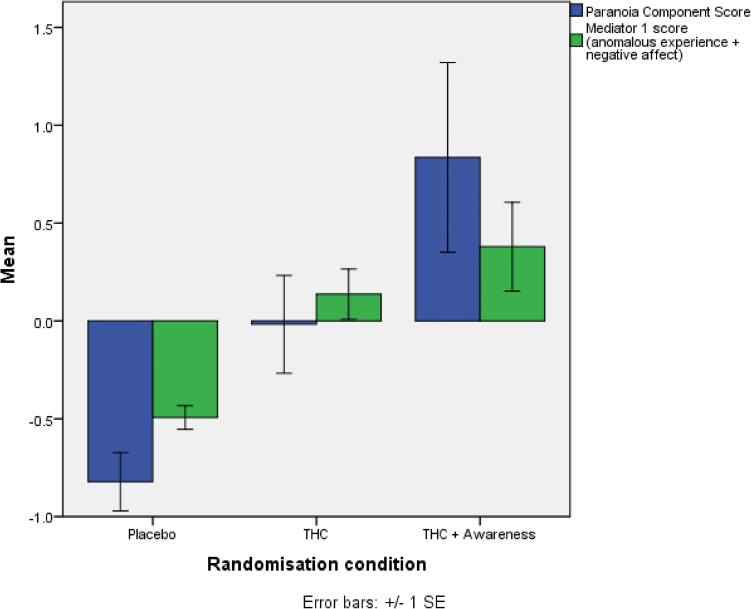
Composite scores for paranoia and the main mediator by randomization group.

An ANCOVA with the paranoia principal component as the dependent variable, and controlling for baseline paranoia and anxiety levels, showed that THC significantly increases paranoia, THC coefficient = 0.91, SE = 0.43, *t* = 2.15, *P* = .034, and that the cognitive awareness condition, if it actually has any effects, may increase paranoia but not significantly, Cognitive Awareness coefficient = 0.51, SE = 0.44, *t* = 1.16, *P* = .247 (see figure 2). Controlling for previous cannabis use did not affect the findings. (see supplementary table S2 for individual paranoia outcome scores). Consistent with the analysis, there was a significant effect on self-reported psychotic symptoms as assessed by the CAPE (controlling for baseline anxiety and paranoia), THC coefficient = 4.20, SE = 1.19, *t* = 3.54, *P* = .001, Cognitive Awareness coefficient = 1.02, SE = 1.23, *t* = 0.83, *P* = .407.

### Effects on Mediators

ITT effects of the randomization condition on the mediation variables are reported in table 2. THC significantly increased scores on component 1 (anomalous experiences and negative affect) and decreased scores on component 3 (working memory). The cognitive awareness condition decreased component 8 (catastrophizing and jumping to conclusions).

**Table 2.** Intention-to-Treat Effects on the Mediators

	Coefficient	SE	*t*	*P* Value
Component 1 (Anomalous experiences and negative affect)				
THC	0.62	0.21	2.95	.004**
Cognitive Awareness	0.14	0.22	0.66	.512
Component 2 (Self-focus)				
THC	0.24	0.22	1.12	.267
Cognitive Awareness	0.10	0.23	0.43	.667
Component 3 (Working memory)				
THC	−0.49	0.21	−2.39	.019*
Cognitive Awareness	−0.33	0.21	−1.55	.125
Component 4 (External attention)				
THC	0.01	0.23	0.03	.974
Cognitive Awareness	−0.15	0.23	−0.61	.544
Component 5 (Interpersonal sensitivity)				
THC	−0.40	0.22	−1.81	.073
Cognitive Awareness	0.15	0.23	0.63	.531
Component 6 (Belief flexibility and positive beliefs)				
THC	0.42	0.22	1.88	.063
Cognitive Awareness	−0.42	0.22	−1.84	.069
Component 7 (Threat anticipation)				
THC	−0.17	0.23	−0.74	.459
Cognitive Awareness	0.08	0.24	0.35	.726
Component 8 (Catastrophizing and JTC)				
THC	0.02	0.22	0.08	.935
Cognitive Awareness	−0.59	0.23	−2.56	.012*

*Note:* JTC, jumping to conclusions; THC, Δ_9_-tetrahydrocannabinol. *P < .05, **P < .01.

### Mediation Analysis

Table 3 shows the results when the ITT effect for randomization condition on paranoia includes each mediator in turn. The key tests are for components 1 and 3, for which ITT effects on these mediators have been established. It can be seen that there is full mediation for component 1 (anomalous experiences and negative affect), since the effect of THC on paranoia becomes nonsignificant but the effect of component 1 is highly significant. Consistent with this finding, there is little evidence of mediation by component 3 (working memory).

**Table 3.** Effect on Paranoia of Randomization Condition and the Mediators Entered Together in Regression Analyses

	Coefficient	SE	*t*	*P* Value
THC	−0.14	0.32	−0.43	.667
Cognitive Awareness	0.46	0.32	1.42	.158
Component 1 (Anomalous experiences and negative affect)	1.47	0.14	10.46	<.001***
THC	0.71	0.44	1.62	.109
Cognitive Awareness	0.64	0.45	1.42	.157
Component 2 (Self-focus)	0.26	0.19	1.34	.182
THC	0.76	0.45	1.68	.096
Cognitive Awareness	0.66	0.46	1.43	.155
Component 3 (Working memory)	−0.03	0.20	−0.14	.887
THC	0.77	0.43	1.78	.077
Cognitive Awareness	0.72	0.45	1.62	.109
Component 4 (External attention)	0.37	0.18	2.07	.041*
THC	0.89	0.44	2.02	.045*
Cognitive Awareness	0.63	0.45	1.39	.168
Component 5 (Interpersonal sensitivity)	0.30	0.19	1.60	.112
THC	0.79	0.45	1.76	.081
Cognitive Awareness	0.66	0.46	1.42	.160
Component 6 (Belief flexibility and positive beliefs)	−0.03	0.19	−0.18	.858
THC	0.77	0.44	1.75	.084
Cognitive Awareness	0.67	0.46	1.47	.144
Component 7 (Threat anticipation)	−0.01	0.19	−0.06	.951
THC	0.77	0.44	1.75	.082
Cognitive Awareness	0.75	0.47	1.60	.112
Component 8 (Catastrophizing and JTC)	0.14	0.19	0.72	.472

*Note:* JTC, jumping to conclusions; THC, Δ_9_-tetrahydrocannabinol. *P < .05, ***P < .001.

## Discussion

Paranoia is a key psychotic experience, distributed as a quantitative trait in the population, which requires explanation in its own right. This is the first experimental study testing the causal effects of THC on paranoia specifically, and it also determined the mechanism of action, taking advantage of the 90-minute period for which the drug was active in participants. It included an extensive assessment battery of paranoia, using a real life social situation, an immersive virtual reality test, self-report questionnaires, and an interviewer assessment. The study clearly establishes that THC causes paranoia in vulnerable individuals.

THC also led to the occurrence of anomalous experiences, anxiety, worry, depression, and negative thoughts about the self. These factors—highly plausible candidates derived from a theoretical model—fully explained the increase in paranoia. This is evidence for their role in causing paranoia. The clear clinical implication is that reducing negative emotion in patients with delusions, eg, by reducing the tendency to worry, testing out anxious fears, and increasing self-confidence, will lead to improvements in paranoia.^4^ Also, the identification, normalization, and reduction of subtle anomalies of experience (eg, by reducing triggers and learning to tolerate the confusing sensory experiences) are clinically warranted. It is intriguing that negative affect and anomalous experience were so closely tied together. As in previous studies, THC also impaired working memory,^14^ but there was no evidence that changes in working memory were responsible for the occurrence of paranoia.

There is a note of caution: the validity of the mediation analysis is dependent on the assumption that an increase in component 1 (anomalous experiences, anxiety, worry, depression, and negative thoughts about the self) is the intermediate variable on the pathway from THC administration to increases in paranoia. The data cannot exclude the possibility that the change in paranoia leads to the change in component 1. However, if we fit a regression model for component 1 with paranoia, experimental condition and baseline levels of paranoia and anxiety as covariates, we obtain a less parsimonious result. Although there is a highly significant effect of paranoia on component 1 (estimated regression coefficient: +0.342 with SE = 0.033; *t* = 10.46; *P* < .001), there remains an unexplained effect of THC that is not explained by paranoia (+0.355 with SE = 0.152; *t* = 2.34; *P* = .021). We conclude from both a theoretical perspective and the relative simplicity of the first model that paranoia is likely to be the distal outcome.

By the inclusion of an awareness condition, the study also made the first experimental attempt to block the misinterpretation considered central to paranoia. Contrary to prediction, this psychological manipulation did not decrease paranoia but perhaps had a paradoxical effect of exacerbating it, though statistical significance was not reached. The awareness condition may have increased sensitivity to paranoid thoughts. Interestingly, some participants in this condition reported being aware that their suspiciousness was due to having had the cannabis, ie, awareness affected judgments of the cause of such fears. As one participant in the awareness condition put it: “I’m always a bit sensitive but this intensified totally because I’m full of cannabis.” The study indicates that misinterpretations in paranoia do not shift readily simply with provision of an alternative verbal explanation and that unintended consequences could arise from this approach.

The study could not address several issues. It is impossible to rule out biases in the estimation of direct and indirect effects arising from hidden confounding. There would have been benefits for learning about the effects of the psychological manipulation by including an additional condition in which the cognitive awareness training was also provided with the placebo; however, adding further randomization conditions causes practical difficulties since recruitment was labor intensive, with almost 2000 people being screened for participation. Arguably, greater effects would have been observed by increasing the dose of THC, though attrition from side effects would have compromised data collection. The dose had ecological validity since it is equivalent to about one strong cannabis cigarette. It would also have strengthened causal claims to have included randomization to other constitutes of cannabis, such as cannabidiol,^18^ which may show opposite effects on the mechanisms underlying paranoia. The statistical strategy of PCA lessens the problem of multiple testing, which is a difficulty for the evaluation of multifactorial models, but does not provide results for individual assessments. The conclusions need to be validated by further experiments. As experiences such as paranoia begin to receive research attention in their own right, we expect to see the emergence of similar experimental studies that determine the causes underlying individual psychotic experiences.

## Supplementary Material

Supplementary material is available at http://schizophre niabulletin.oxfordjournals.org.

## Funding

This work was supported by a UK Medical Research Council (MRC) Senior Clinical Fellowship (G0902308) awarded to D.F. P.D.M. is part funded by the National Institute for Health Research (NIHR) Biomedical Research Centre at South London and Maudsley NHS Foundation Trust and King’s College London.

## Supplementary Material

Supplementary Data
