# Supplementary material for: How Cannabis Causes Paranoia: Using the Intravenous Administration of ∆9-Tetrahydrocannabinol (THC) to Identify Key Cognitive Mechanisms Leading to Paranoia
Source: Schizophr Bull. 2014 Jul 16;41(2):391–9. doi: 10.1093/schbul/sbu098 (PMC4332941; doi:10.1093/schbul/sbu098)
Supplement: Supplementary Data [file supp_41_2_391__index.html]

How Cannabis Causes Paranoia: Using the Intravenous Administration of ∆9-Tetrahydrocannabinol (THC) to Identify Key Cognitive Mechanisms Leading to Paranoia — How Cannabis Causes Paranoia: Using the Intravenous Administration of ∆9-Tetrahydrocannabinol (THC) to Identify Key Cognitive Mechanisms Leading to Paranoia — Supplementary Data 

# How Cannabis Causes Paranoia: Using the Intravenous Administration of ∆9-Tetrahydrocannabinol (THC) to Identify Key Cognitive Mechanisms Leading to Paranoia

## Supplementary Data

Data files

**Files in this Data Supplement:**

- Supplementary Data - Supplementary Data
