# Supplementary material for: How Cannabis Causes Paranoia: Using the Intravenous Administration of ∆9-Tetrahydrocannabinol (THC) to Identify Key Cognitive Mechanisms Leading to Paranoia
Source: Schizophr Bull. 2014 Jul 16;41(2):391–9. doi: 10.1093/schbul/sbu098 (PMC4332941; doi:10.1093/schbul/sbu098)
Supplement: Supplementary Data [file supp_sbu098_Supplementary_Materials.doc]

Supplementary Figure 1. The virtual train at the station.


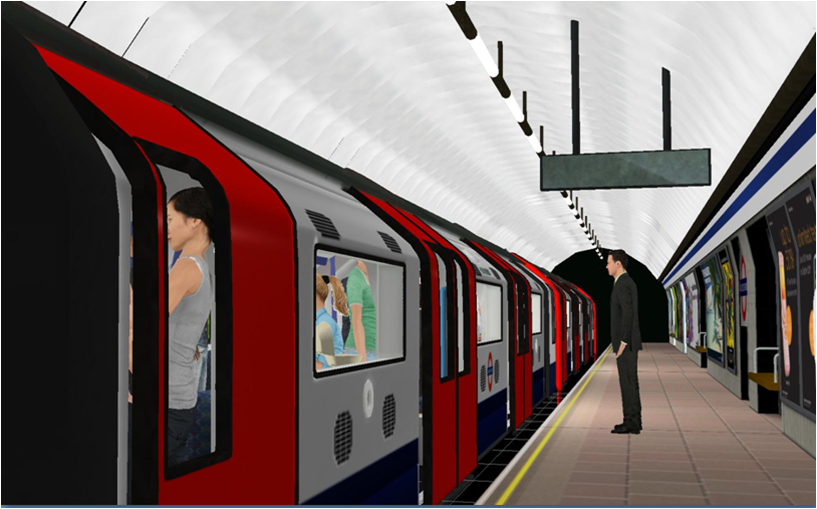


Figure 1b. The virtual train carriage


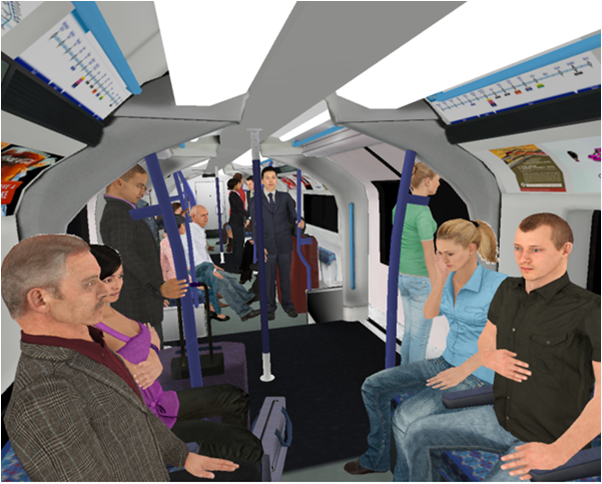


Figure 1c. The virtual reality laboratory


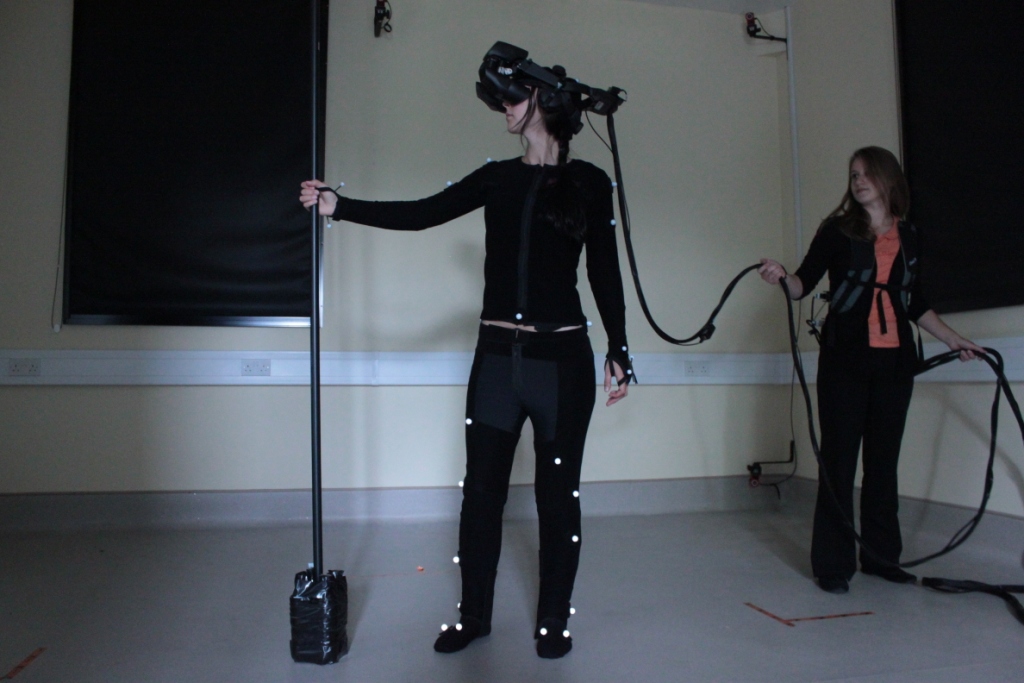


Supplementary Table 1

| **Principal Components Analysis of mediators – Varimax rotated component matrix** | | | | | | | | |
| --- | --- | --- | --- | --- | --- | --- | --- | --- |
|  | Component | | | | | | | |
|  | 1 | 2 | 3 | 4 | 5 | 6 | 7 | 8 |
| Anomalous perceptions total post | .688 |  |  |  |  |  |  |  |
| Anxious post | .807 |  |  |  |  |  |  |  |
| Anxious at the end of the study | .780 |  |  |  |  |  |  |  |
| Anxiety post (BAI) | .747 |  |  |  |  |  |  |  |
| Anxious thoughts in VR | .701 |  |  |  |  |  |  |  |
| Worried post | .806 |  |  |  |  |  |  |  |
| Worried at the end of the study | .827 |  |  |  |  |  |  |  |
| Worry steps post |  |  |  |  |  |  |  | .739 |
| Inner thoughts focus post |  | .774 |  |  |  |  |  |  |
| Inner thoughts focus at the end of the study |  | .691 |  |  |  |  |  |  |
| Appear to others focus post |  | .755 |  |  |  |  |  |  |
| Appear to others focus at the end of the study |  | .694 |  |  |  |  |  |  |
| Surroundings focus post |  |  |  | .821 |  |  |  |  |
| Surroundings focus at the end of the study |  |  |  | .776 |  |  |  |  |
| Sad post | .703 |  |  |  |  |  |  |  |
| Sad at the end of the study | .783 |  |  |  |  |  |  |  |
| Interpersonal sensitivity post |  |  |  |  | .692 |  |  |  |
| Threat anticipation post |  |  |  |  |  |  | .764 |  |
| Negative self beliefs post | .611 |  |  |  | .419 |  |  |  |
| Positive self beliefs post |  |  |  |  |  | .684 |  |  |
| Negative others beliefs post |  |  |  | .479 |  |  |  | .580 |
| Positive others beliefs pre |  |  |  |  | .437 | .502 | -.490 |  |
| Digit span forward post |  |  | .808 |  |  |  |  |  |
| Digit span backward post |  |  | .764 |  |  |  |  |  |
| Letter-Number post |  |  | .814 |  |  |  |  |  |
| JTC Draws to decision post |  |  |  |  |  |  | .429 | -.494 |
| Belief flexibility-post |  |  |  |  |  | -.723 |  |  |

Supplementary Table 2. Post vial administration scores for the individual paranoia outcomes

|  | **Placebo (n = 41)**  **Mean (SD)** | **THC (n = 41)**  **Mean (SD)** | **THC and Awareness (n = 39)**  **Mean (SD)** |
| --- | --- | --- | --- |
| **Paranoid Visual Analogue Scales after randomisation** | 13.9 (20.7) | 34.5 (45.2) | 60.9 (102.6) |
| **Paranoid Visual Analogue Scales at end of testing session** | 13.9 (27.5) | 27.7 (36.0) | 40.2 (66.9) |
| **Paranoia on walk (visual analogue scales)** | 11.5 (23.0) | 43.8 (48.6) | 69.3 (87.7) |
| **Paranoia in virtual reality (SPSS score)** | 11.1 (2.2) | 12.6 (4.6) | 14.1 (8.1) |
| **Paranoia in virtual reality (hostility of computer characters)** | 6.8 (9.8) | 15.6 (17.3) | 17.8 (27.8) |
| **PANSS suspiciousness score** | 1.4 (0.7) | 2.0 (1.1) | 2.3 (1.2) |
